# A Call for Urgent Monitoring of Food and Water Security Based on Relevant Indicators for the Arctic

**DOI:** 10.1007/s13280-013-0427-1

**Published:** 2013-08-06

**Authors:** Lena Maria Nilsson, Georgia Destouni, James Berner, Alexey A. Dudarev, Gert Mulvad, Jon Øyvind Odland, Alan Parkinson, Constantine Tikhonov, Arja Rautio, Birgitta Evengård

**Affiliations:** 1 Arctic Research Centre, Umeå University, Umeå, Sweden; 2 Nutritional Research, Department of Public Health and Clinical Medicine, Umeå University, 901 85 Umeå, Sweden; 3 Department of Physical Geography and Quaternary Geology and Bert Bolin Centre for Climate Research, Stockholm University, 106 91 Stockholm, Sweden; 4 Division of Community Health, Alaska Native Tribal Health Consortium, Anchorage, AK USA; 5 Hygiene Department, Northwest Public Health Research Center, 4, 2-Sovetskaya Street, 191036 St. Petersburg, Russia; 6 Greenland Center for Health Research, University of Greenland, Postboks 1001, 3900 Nuuk, Greenland; 7 Faculty of Health Sciences, University of Tromsø, 9019 Tromsö, Norway; 8 Arctic Investigations Program, US Centers for Disease Control & Prevention, Anchorage, AK 99516 USA; 9 Environmental Public Health Division, First Nations and Inuit Health Branch, Health Canada, Ottawa, ON Canada; 10 Thule Institute, University of Oulu, P.O. Box 7300, Oulu, Finland; 11 Division of Infectious Diseases, Department of Clinical Microbiology, Umeå University Hospital, 901 85 Umeå, Sweden

**Keywords:** Food security, Water security, Arctic, Circumpolar, Climate change, Public health

## Abstract

This perspective paper argues for an urgent need to monitor a set of 12 concrete, measurable indicators of food and water security in the Arctic over time. Such a quantitative indicator approach may be viewed as representing a reductionist rather than a holistic perspective, but is nevertheless necessary for actually knowing what reality aspects to monitor in order to accurately understand, quantify, and be able to project critical changes to food and water security of both indigenous and non-indigenous people in the Arctic. More relevant indicators may be developed in the future, taking us further toward reconciliation between reductionist and holistic approaches to change assessment and understanding. However, the potential of such further development to improved holistic change assessment is not an argument not to urgently start to monitor and quantify the changes in food and water security indicators that are immediately available and adequate for the Arctic context.

## Introduction

Changes in climate and land–water use, together with socioeconomic and industrial factors, may have serious impacts on food and water security worldwide (FAO 2012; Destouni et al. 2013). Because of complex cause-effect and feedback systems, global climate change has been most and earliest noticeable in the Arctic (Post et al. 2009). Animals and humans living in the region are already affected, and will continue to be affected in various ways, both in remote areas and in areas with more developed infrastructure (AMAP 2009). By extension, this means that food and water security may be threatened for people and animals in Alaska, Greenland, and Iceland, and the northernmost parts of Norway, Sweden, Finland, Russia, and Canada. Many indigenous groups in these areas live close to nature, and are even more vulnerable than other humans (Chatwood et al. 2012). For example in Nunavut, Canada, nearly 70 % of the Inuit preschoolers have been found to reside in food-insecure households (Egeland et al. 2010), 45 % of the indigenous people of Chukotka, Russia, lack bath or shower in their homes (SLiCA 2012), and in Fennoscandia reindeer herding, an important pre-requisite for the traditional Sami food culture, is threatened by climate change (Laaksonen et al. 2010). Together with an increasing political and economical interest for the resources of the North, the region may sooner or later be facing further resilience challenges with regard to food and water security for vulnerable indigenous population groups.

Food and water security could be described from at least two different scientific perspectives: a holistic and a reductionistic. The common starting point for both these approaches is the fact that living beings interacting with environment is a complex system. The reductionist method, introduced by Descartes (scientist 1596–1650), seeks to break down the complex system into fragmented parts, in order to be able to study each part individually, and then draw conclusions about the over-all system. Contrary reality, from a holistic perspective, should best be described with a system approach as a whole, not as a sub-set of fragmented parts. Both these approaches have their advantages and disadvantages, and have been extensively discussed among scientists for decades (e.g., Ahn et al. 2006; Greek and Rice 2012; McGinley 2012).

In January 2013, an international joint monitoring project for food and water security in an Arctic health context was proposed to the Arctic Council’s Sustainable Development Working Group (SDWG), an intergovernmental forum for Arctic governments and peoples. The aim of the project was to identify and, as needed, develop improved monitoring programs for a set of relevant indicators of food and water security. The identification of relevant indicators was done by mainly focusing on already existing national registries and bio-monitoring within the eight Arctic countries, as basis for further harmonization of national data for making them scientifically comparable (Nilsson and Evengård 2013), and based on the existing WHO and FAO indicators.

The present perspective paper aims at further elucidating and expanding on the work and argumentation presented in the food and water security monitoring project report (Nilsson and Evengård 2013), in order to open and widen the important discussion on the need and relevance of indicator monitoring for food and water security in the Arctic. In particular, the paper presents and argues for the needs of reductionist perspectives, in addition to more holistic perspectives, which have been warranted by some non-governmental organizations in this context, and to the links between and complementary aspects of the two perspectives. A parallel paper, describing the methods and rationale for the indicator selection has been submitted to another journal, as a more concrete base for future descriptive and quantitative publications (Nilsson et al., unpublished).

## Indicators of Food and Water Security

Food and water security indicators may be defined as summary measures of one or more of the dimensions of change in food and water security, or of the result of a program activity aimed at improving food and water security for a target population (Glacken 2009; Nilsson and Evengård 2013). In other words, the purpose of an indicator is to quantify an observable summary measure or a target component, which can reflect changes of wider and multi-causal effects. Even if this may be viewed as a reductionist perspective on change, a large number of food and water security indicators have indeed been considered and used in various contexts (Hoddinott 1999). For example, until 2010, food security in the world has been described by the World Health Organization (WHO) mainly by two indicator measures: undernourishment and per person dietary energy supply (DES) (FAO 2012). Furthermore, since 2012 the FAO uses a set of more than 20 different food security indicators (FAO 2012). Such indicators could be used for monitoring in the Arctic, as in other parts for the world.

Food and water security indicators can be divided into two main groups: direct measures and indirect measures. Direct measures include various methods to describe actual food or water intake on individual or household level, perceptions of food and security related to seasonal shortfalls, and cultural acceptability of foods (Glacken 2009). Indirect measures include factors that may be associated with food or water security, such as household size/composition, sources of household income, access to credit, sale of assets and food stores (Glacken 2009). Some of the indicators proposed for monitoring food and water security in the Arctic Health context (9) were direct and indirect. All of them had been used previously to measure different dimensions of food and water security in a circumpolar context, i.e., all of them were indicators that had already been considered relevant for the Arctic and were possible to identify and to some degree quantify through a literature search. From an initial list of 20 potential indicators, the following 12 main indicators were chosen and proposed (9):

1. *Healthy weight* Measured on a population level as body mass index (kg/m^2^) or proportion of the population being obese (BMI > 30). These measures are likely abundantly collected at least in children all over the circumpolar area.
2. *Self-estimated proportion of traditional food in diet* Measured by questionnaire on a household or population level as self-estimated proportion of traditional food in diet or proportion of the individuals that ate traditional food the previous day or week. These measures reflect the importance of traditional food security as a component of food security.
3. *Non*-*monetary food accessibility* Measured as proportion of the households or families that have eligible hunters, fishers, collectors, or herders. In previous studies, only eligible hunters have been recognized as a potential indicator of food security (AMAP 2009). None of these four measures are monitored at present, but were considered relevant indicators with all the advantages and disadvantages of survey data.
4. *Monetary food accessibility* Measured as cost of a nutritious food basket in relation to disposable household income. This measure was considered a high information value to a low monitoring cost and a comparable, practical, and potentially standardized measure.
5. *Foodborne diseases* Measured as incidence rates in men and women and seroprevalence in men and women and in subsistence species. Though under-diagnosis may exist, data on incidence rates in humans are continuously collected in most Arctic areas. Seroprevalence in human and subsistence species may be more expensive, and is at present not monitored on a regular basis.
6. *Food-related contaminants* Measured as chemical contaminants in food, biological contaminants in food, and chemical contaminants in human tissue. With exception of biological contaminants, these indicators are already largely covered by the Arctic Monitoring and Assessment Program’s Human Health Assessment Group (AMAP/HHAG). Since data are collected continuously in many countries, these measures are widely available, but the importance of collaborating with AMAP should be stressed.
7. *Per capita renewable water* Measured as m^3^ capita^−1^ year^−1^. The abundant water supplies within the Arctic area are not always available to consumers because of problems of water quality and timing of availability (Young et al. 2012), but this measure can help monitor important changes linked to climate change.
8. *Accessibility of running water* Measured as proportion of the population having access to running water. This is a widely used indicator with a high information value to a low monitoring cost.
9. *Waterborne diseases* Measured as incidence rates and seroprevalence in men and women. Incidence data are already continuously collected, and seroprevalence may be monitored simultaneously with seroprevalence of foodborne diseases.
10. *Drinking water contaminants* Measured as chemical contaminants in drinking water exceeding national threshold levels, occasions when consumers have been recommended to boil their drinking water and microbiological contaminants in drinking water exceeding national threshold levels. Both number of exceeding occasions and proportional values are of importance. Data are already continuously collected nationwide in all Arctic countries.
11. *Authorized water quality assurance* Measured as proportion of the population that has access to water sources within the authorities’ water quality control. An overview is needed, as there is a risk that there are too diverse water systems in different areas of the Arctic to make it suitable for international comparisons. This measure provides a relatively high information value to a relatively low cost.
12. *Water safety plans* Measured as the presence of compulsory water safety plan according to the WHO’s standard (WHO 2005). Water safety plan is a tool for securing the entire chain from raw water to the pipes and includes related concepts such as the risk assessment tool hazard analysis and critical control points (HACCP) and raw water protection. This measure is another example of a tool that provides a relatively high information value to a relatively low cost.

## Indigenous, Holistic, and Quantitative Monitoring Perspectives

From an indigenous perspective, availability of traditional food is a core issue regarding food security, since traditional food is an essential element of culture and an important source of nutrients. In some rural areas in the Arctic it is the only food available. Thus, in a recent report from Canada, it was concluded that access to both market food and traditional food must be considered in food security assessments (Egeland et al. 2011). For example in Chukotka, in the Russian Arctic, locally harvested food is of fundamental value (in some remote settlements—almost the only food source) for indigenous peoples; both coastal and inland indigenous people consume on average about 170 kg of traditional food per person per year (Dudarev 2012). In Greenland, marine mammals are considered a staple in traditional cuisine, but since these species have been polluted by industrial contaminants in modern time, dietary guidelines have to be updated continuously to provide a balance between imported and traditional food (Bjerregaard and Mulvad 2012).

While more studies are needed to identify the multi-faceted dimensions of food and water security in Arctic indigenous communities, some assessment of traditional food and water security were considered already in our selection of food and water security indicators. These included the self-estimated proportion of traditional food in diet (number 2), non-monetary food accessibility (number 3), and accessibility of running water (number 8), previously included in the SLiCA study (a survey of living conditions in the Arctic: Inuit, Saami, and the Indigenous Peoples of Chukotka) (Eliassen et al. 2012). Such data will prove valuable for indigenous communities, providing quantitative data on food and water security for action at local, national, and governmental levels.

With regard to holistic perspective on Arctic change, a Pubmed search on “Arctic + holistic” with a filter for research on humans rendered only two relevant papers (Bird et al. 2008; Lewis 2011), both of them dealing with human experiences of different kinds of physiological processes affecting the human bodies (aging and suffering from diabetes). Furthermore, much of existing indigenous knowledge and perspectives on Arctic change remain unpublished and should be further explored. This kind of in-depth human knowledge is of great value from cognitive and philosophical perspectives, but may be of lesser value as a tool for quantitative assessment and monitoring of change. While Arctic peoples’ experiences of changes in food and water security should be explored from a holistic perspective, the monitoring of actual ongoing change requires also quantitative assessments. Ongoing quantification of actual indicator change would lay a foundation for future research and monitoring, as well as enable the development of forecasts for potential impacts of such changes on indigenous and non-indigenous people in the Arctic.

Relevant follow-up studies of actual change to measurable (quantifiable) food and water security indicators in addition to providing opportunities for expression of Indigenous perspectives on changes occurring across the Arctic can strengthen the link and complementarity of the two perspectives, leading to improved change detection, assessment, mitigation, and adaptation for the benefit of all Arctic peoples and societies.

## Identifying Knowledge Gaps

Changes in the Arctic are expected to impact food and water security, particularly taking into account the projected impacts of future climate change (Kattsov et al. 2005; McBean et al. 2005).

There is a need of a monitoring system that is able to visualize and quantify these changes, especially regarding availability, accessibility, and safety of traditional and local staple foods that are affected by local climate change. The proposed indicators: self-estimated proportion of traditional food in diet (number 2), non-monetary food accessibility (number 3), foodborne diseases (number 5), and food-related contaminants (number 6) will be of most importance from this perspective, though it should be noted that traditional food may also be available within the monetary food system measured by the proposed indicator, monetary food accessibility (number 4). However, the monitoring system should also regard availability, accessibility and safety of market food, for which cost and quality may be affected by climate changes outside the Arctic area as well as by future political and economical changes worldwide. For example, in Sweden, a national early warning system for outbreak surveillance of food- and waterborne diseases by telephone triage has recently been evaluated and promoted by scientists (Andersson et al. 2013). Decreasing consumption of traditional food because of decreasing stocks, as well as environmental and biological contaminants, could be a driver of the already ongoing nutritional transition toward a Westernized dietary pattern, reflected by the proposed indicator, healthy weight (number 1), and an increased dependency on monetary achieved food reflected by the proposed indicator, monetary food accessibility (number 4).

Freshwater-related changes in the Arctic include increasing river flows (Peterson et al. 2002; Peterson et al. 2006; Dyurgerov et al. 2010) and increasing groundwater contribution to those flows (Smith et al. 2007; Lyon and Destouni 2010), shorter extent of snow cover season (Brown et al. 2010; Callaghan et al. 2011), increased meltwater flows from glacier mass loss (Kaser et al. 2006; Dyurgerov et al. 2010), and permafrost degradation that in turn changes groundwater flow into streams and rivers (White et al. 2007; Lyon et al. 2009; Lyon and Destouni 2010; Frampton et al. 2011).

In order to accurately assess and plan for adaptation to such Arctic water changes, hydrological and hydrochemical monitoring systems are required that can provide adequate change information, for instance with regard to the proposed water indicators, per capita renewable water (number 7) and water-related contaminants (number 10). Accurate information about these indicators constitutes in turn also a necessary basis for relevant development of other important water indicators: accessibility of (good quality) running water (number 8), authorized water quality assurance (number 11), and water safety plans (number 12). However, in contrast to these needs, Arctic water observation systems have been in decline during recent decades (Hinzman et al. 2005; Walsh et al. 2005), and there is severe lack of long-term and accessible water quality and chemistry data for large parts of the Arctic (Bring and Destouni 2009) and large inaccuracy in climate model projections of Arctic precipitation (Bring and Destouni 2011). As a consequence, quite different conclusions arise about what constitutes rational water monitoring priorities when considering different data, and system/change perspectives (Bring and Destouni 2013).

## Conclusion

In conclusion, with effects of climate change being especially dramatic in the Arctic, there is an urgent need to monitor changes in food and water security. This is the basis for the 12 indicators proposed by the AHHEG/SDWG and the AMAP/HHAG in January 2013 (Nilsson and Evengård 2013). This indicator approach would enable the development of initial datasets comparable between different countries and worldwide and provide quantitative data on food and water security for local, national, and international governmental levels.

The reductionist quantitative indicator perspective is necessary for initiating comparable food and water security monitoring, and for accurately understanding ongoing changes and being able to project future changes. While more indicators may be developed in the future, that may include indicators that are derived from studies of food and water security from the indigenous perspective, the development of these 12 quantitative indicators will allow initiation of studies to assess food and water security in the Arctic.
